# Motion-based super-resolution in the peripheral visual field

**DOI:** 10.1167/17.9.15

**Published:** 2017-08-24

**Authors:** Jonathan A. Patrick, Neil W. Roach, Paul V. McGraw

**Affiliations:** nwr@psychology.nottingham.ac.u; pvm@psychology.nottingham.ac.uk; School of Optometry, University of California, Berkeley, Berkeley, CA, USA; Nottingham Visual Neuroscience, School of Psychology, The University of Nottingham, Nottingham, UK; Nottingham Visual Neuroscience, School of Psychology, The University of Nottingham, Nottingham, UK

**Keywords:** motion, psychophysics, resolution

## Abstract

Improvements in foveal acuity for moving targets have been interpreted as evidence for the ability of the visual system to combine information over space and time, in order to reconstruct the image at a higher resolution (super-resolution). Here, we directly test whether this occurs in the peripheral visual field and discuss its potential for improving functional capacity in ocular disease. The effect of motion on visual acuity was first compared under conditions in which performance was limited either by natural undersampling in the retinal periphery or by the presence of overlaid masks with opaque elements to simulate retinal loss. To equate the information content of moving and static sequences, we next manipulated the dynamic properties of the masks. Finally, we determined the dependence of motion-related improvements on the object of motion (target or mask) and its trajectory (smooth or jittered). Motion improved visual acuity for masked but not unmasked peripheral targets. Equating the information content of moving and static conditions removed some but not all of this benefit. Residual motion-related improvements were largest in conditions in which the target moved along a consistent and predictable path. Our results show that motion can improve peripheral acuity in situations in which performance is limited by abnormal undersampling. These findings are consistent with the operation of a super-resolution system and could have important implications for any pathology that alters the regular sampling properties of the retinal mosaic.

## Introduction

The ability to recognize spatial detail such as words and letters in the visual field is usually quantified in terms of acuity. Spatially demanding tasks such as reading are performed using the most sensitive region of the visual field, the fovea. The resolution limit of the fovea is set by the transfer function of the eye's optical apparatus (Jennings & Charman, 1981; Williams, Artal, Navarro, McMahon, & Brainard, 1996). However, with increasing retinal eccentricity, acuity deteriorates in line with changes to the sampling density of retinal circuits (Curcio, Sloan, Kalina, & Hendrickson, 1990; Curcio, Sloan, Packer, Hendrickson, & Kalina, 1987; Rossi & Roorda, 2010). Therefore, resolution becomes sampling limited in the peripheral visual field (Anderson & Hess, 1990; Anderson & Thibos, 1999). As a result, spatial frequencies beyond the resolution limit are detected but appear highly distorted (Thibos, Still, & Bradley, 1996; Thibos, Walsh, & Cheney, 1987). Although foveal vision is limited by optical factors, aliases can also be generated in the fovea if the blurring properties of the eye's optics are circumvented (Williams, 1985).

**Figure 1:**
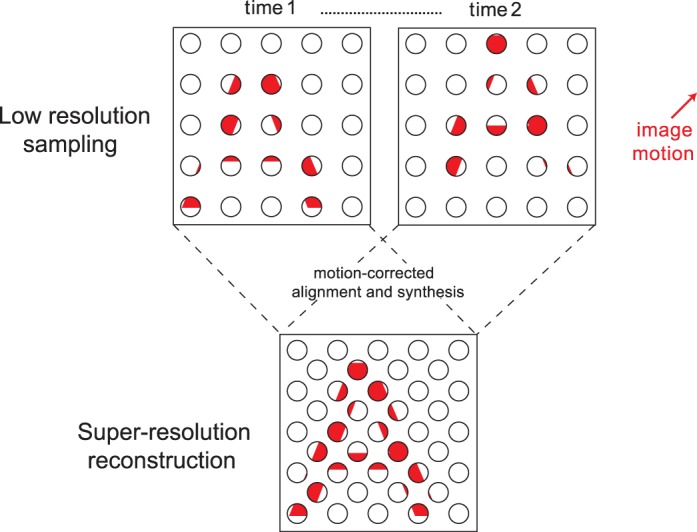
When an image moves slowly across a receptor array, multiple low-resolution samples obtained at different times can be synthesized to reconstruct a more detailed image.

In digital imaging systems, sampling limits can be overcome to some extent by super-resolution (SR) techniques that exploit small motion-induced shifts in an image to reconstruct it at a higher resolution (Park, Park, & Kang, 2003). The principle behind this process is illustrated in Figure 1. Low-resolution images obtained at successive points in time (top row) are motion corrected and merged to form a single image with much greater spatial detail (bottom row). This form of image analysis is thought to operate in the visual system of certain species of jumping spider (salticids), where gaze is initially stabilized on an object of interest and followed by a series of small-amplitude retinal oscillations. This scanning process allows the spider to generate a series of similar images that can be used to synthesize a higher resolution facsimile of the object. As a result, the spider is able to make much finer spatial discrimination judgments than would normally be supported by the properties of its receptor array (Jackson & Harland, 2009; Land, 1969a, 1969b).

SR processing is now widely employed in a range of real-world applications (e.g., medical imaging, high-definition photography, military surveillance). Despite this, its wider role in biological visual systems remains largely unexplored. In human vision, there is some evidence to suggest that motion aids the resolvability of spatial patterns viewed through apertures (Nishida, 2004; Stappers, 1989) or occluded by opaque masks (Frisén, 2010; Kellman, Yin, & Shipley, 1998; Scholl & Pylyshyn, 1999). To simulate changes in sampling density resulting from pathology of the retinal array, Frisén (2010) measured monocular letter acuity in central vision while superimposing various stationary masks. Whereas static acuity fell systematically with increasing mask density, acuity for moving targets was much less affected. This was interpreted as evidence for SR processing capacity in situations in which acuity is sampling limited. However, because Frisén employed a static mask, a larger number of independent spatial samples of the target were available in moving compared with static conditions. As a result, it is difficult to ascertain whether motion-related improvements in acuity reflect bona fide SR processing or simply the increase in target information available in the stimulus sequence (i.e., probability summation).

Here we describe a series of experiments in which we examine the conditions under which motion improves acuity in the peripheral visual field, to provide a rigorous test of SR processing capacity in human vision.

## Methods

### Participants

Eight observers (mean age = 24.50 years, *SD* = 1.41 years) participated in this study. All had a central acuity level that was equivalent to, or better than, 0 logMAR (20/20, 6/6) measured using an ETDRS acuity chart. Each gave informed consent, and ethics approval was attained from the University of Nottingham School of Psychology Ethics Committee. This study adhered to the tenets of the Declaration of Helsinki.

### Apparatus

Stimuli were generated by PsychoPy version 1.81.01 (Peirce, 2007) on a Mac Mini (late 2012, Apple Inc., Cupertino, CA) and presented on a gamma-corrected 20-in. CRT monitor (LaCie Electron22blueIV, 1,280 × 1,024 resolution; Seagate Technology, Tigard, OR) with a 75-Hz refresh rate (13.3-ms frame duration). Observers sat in a dimly lit laboratory (∼0.5 cd/m^2^) with a chin rest 100 cm from the monitor. At this distance, each pixel subtended 1.05 arcmin of visual angle. Viewing was monocular using the right eye; the contralateral eye was occluded using a standard eye patch. All subjects had sufficient accommodative facility for viewing targets at the test distance of 1 m.

### Stimuli

**Figure 2:**
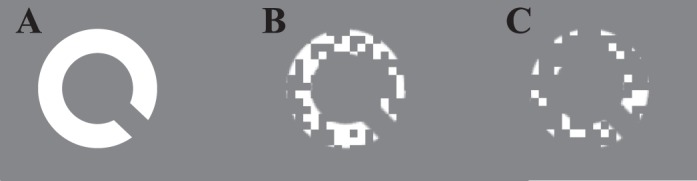
Example images of the target when occluded by the mask. (A) Mask density is set to 0. (B) Mask density is set to 0.5, such that 50% of the mask elements were opaque. (C) Mask density is set to 0.75.

Target stimuli were Landolt Cs created in Sloan font (Pelli, Robson, & Wilkins, 1988). The dimensions of the critical detail of this type of target (the gap) is fixed at 20% of the target diameter. Targets were white (85 cd/m^2^) and presented on a gray background (45 cd/m^2^). Spatial undersampling of the target was simulated by overlaying a 7° × 7° square grid mask, consisting of 5.25 × 5.25 arcmin pixel elements. Depending on the mask density, a proportion of the elements was randomly selected and assigned the same luminance as the background. Examples of masked targets with different densities are shown in Figure 2. A white (85 cd/m^2^) 0.5° × 0.5° fixation cross was presented in the center of the screen, and observers were asked to maintain fixation on this throughout the trial.

### Procedure

On each trial, the target was presented for 0.33 s (25 video frames). A forced-choice orientation discrimination paradigm was employed, whereby participants identified which of the four oblique positions contained the gap in the Landolt C (i.e., lower left, lower right, upper left, upper right). Target and mask dynamics were manipulated across six experimental conditions, which are summarized in Table 1.

**Table 1:**
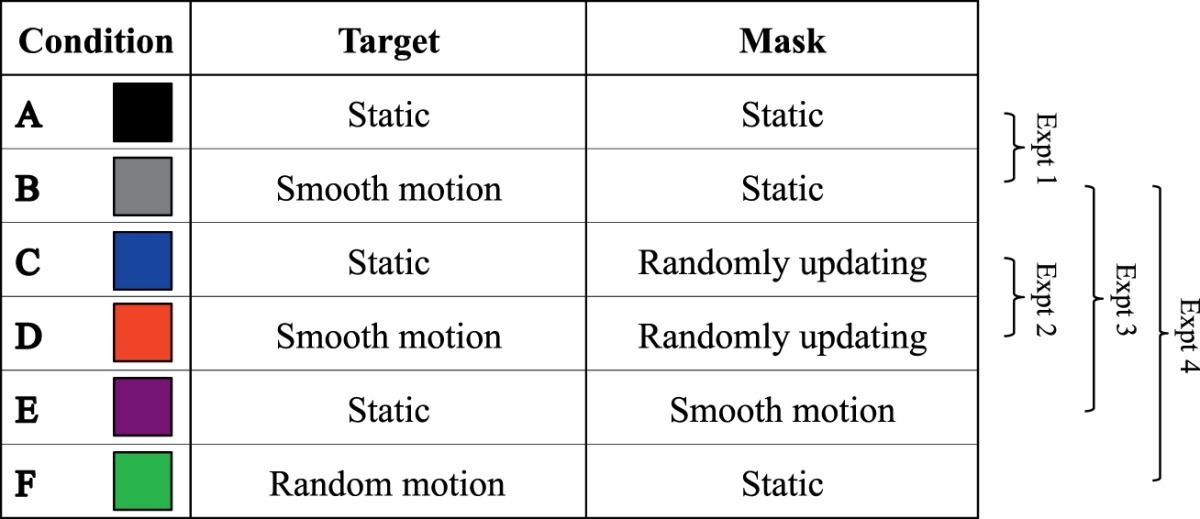
Overview of the experimental conditions. Conditions are color matched to histograms depicting mean size thresholds in Figures 4–7. See main text for descriptions of the different conditions.

**Figure 3:**
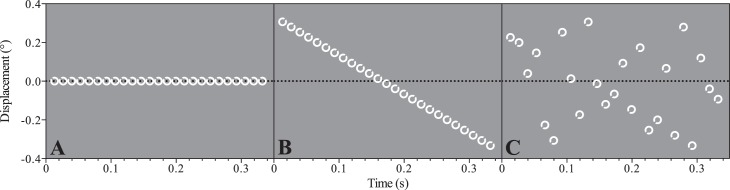
Space-time plots of the motion conditions. Displacement refers to the distance of the target on each frame from a point on the horizontal meridian, 10° to the right of the fixation cross. (A) The target is static. (B) The target is moving sequentially at 2°/s, so is following a smooth path. (C) The target path has been randomized. It can be seen that the individual target locations are identical but are presented in a random order.

- Static target, static mask (see Figure 3A). Target and mask stimuli were centered 10° from fixation along the horizontal meridian in the temporal visual field. The position of both remained fixed throughout the duration of the trial.
- Smooth target motion, static mask (see Figure 3B). Targets moved along an isoeccentric arc (10° eccentricity) at a consistent velocity of 2°/s. Starting and ending positions of the motion path were equally spaced above and below the horizontal meridian. Direction of motion (clockwise/counterclockwise) was randomly assigned on each trial.
- Static target, randomly updating mask. The target and mask remained in a fixed location, but the spatial distribution of mask elements was regenerated on each video frame (i.e., at 75 Hz).
- Smooth target motion, randomly updating mask. As described above, the target moved along an isoeccentric arc at 2°/s, whereas the spatial distribution of mask elements was regenerated on each video frame.
- Static target, smooth mask motion. The mask moved at 2°/s along an isoeccentric arc. The spatial distribution of mask elements remained fixed throughout the trial. Direction of motion (clockwise/counterclockwise) was randomly selected on each trial.
- Random target motion, static mask. Random target motion paths were created by randomizing the order of frame-by-frame spatial coordinates derived from smoothly moving conditions (see Figure 3C). The mask had a fixed spatial configuration and remained in a fixed position throughout the trial.

The target gap size was set to 17.8 arcmin at the beginning of each run, after which it was manipulated via a 3-down 1-up staircase. The staircase had an initial step size of 4.5 arcmin, which halved on every size-increasing reversal. The staircase terminated after eight reversals or 50 trials (whichever came first). Ten runs were carried out for each condition, completed in a random order. Responses were collated across runs and fitted using a maximum likelihood criterion with a logistic function of the form:

\newcommand{\bialpha}{\boldsymbol{\alpha}}\newcommand{\bibeta}{\boldsymbol{\beta}}\newcommand{\bigamma}{\boldsymbol{\gamma}}\newcommand{\bidelta}{\boldsymbol{\delta}}\newcommand{\bivarepsilon}{\boldsymbol{\varepsilon}}\newcommand{\bizeta}{\boldsymbol{\zeta}}\newcommand{\bieta}{\boldsymbol{\eta}}\newcommand{\bitheta}{\boldsymbol{\theta}}\newcommand{\biiota}{\boldsymbol{\iota}}\newcommand{\bikappa}{\boldsymbol{\kappa}}\newcommand{\bilambda}{\boldsymbol{\lambda}}\newcommand{\bimu}{\boldsymbol{\mu}}\newcommand{\binu}{\boldsymbol{\nu}}\newcommand{\bixi}{\boldsymbol{\xi}}\newcommand{\biomicron}{\boldsymbol{\micron}}\newcommand{\bipi}{\boldsymbol{\pi}}\newcommand{\birho}{\boldsymbol{\rho}}\newcommand{\bisigma}{\boldsymbol{\sigma}}\newcommand{\bitau}{\boldsymbol{\tau}}\newcommand{\biupsilon}{\boldsymbol{\upsilon}}\newcommand{\biphi}{\boldsymbol{\phi}}\newcommand{\bichi}{\boldsymbol{\chi}}\newcommand{\bipsi}{\boldsymbol{\psi}}\newcommand{\biomega}{\boldsymbol{\omega}}\[p\left( {correct} \right) = 0.25 + {{0.75} \over {1 + {e^{{{(\mu - x)} \over \sigma }}}}}\]

where *p*(*correct*) is the proportion of correct responses, *x* is the target gap size (arcmin), \newcommand{\bialpha}{\boldsymbol{\alpha}}\newcommand{\bibeta}{\boldsymbol{\beta}}\newcommand{\bigamma}{\boldsymbol{\gamma}}\newcommand{\bidelta}{\boldsymbol{\delta}}\newcommand{\bivarepsilon}{\boldsymbol{\varepsilon}}\newcommand{\bizeta}{\boldsymbol{\zeta}}\newcommand{\bieta}{\boldsymbol{\eta}}\newcommand{\bitheta}{\boldsymbol{\theta}}\newcommand{\biiota}{\boldsymbol{\iota}}\newcommand{\bikappa}{\boldsymbol{\kappa}}\newcommand{\bilambda}{\boldsymbol{\lambda}}\newcommand{\bimu}{\boldsymbol{\mu}}\newcommand{\binu}{\boldsymbol{\nu}}\newcommand{\bixi}{\boldsymbol{\xi}}\newcommand{\biomicron}{\boldsymbol{\micron}}\newcommand{\bipi}{\boldsymbol{\pi}}\newcommand{\birho}{\boldsymbol{\rho}}\newcommand{\bisigma}{\boldsymbol{\sigma}}\newcommand{\bitau}{\boldsymbol{\tau}}\newcommand{\biupsilon}{\boldsymbol{\upsilon}}\newcommand{\biphi}{\boldsymbol{\phi}}\newcommand{\bichi}{\boldsymbol{\chi}}\newcommand{\bipsi}{\boldsymbol{\psi}}\newcommand{\biomega}{\boldsymbol{\omega}}\(\mu \) is the size threshold, and \newcommand{\bialpha}{\boldsymbol{\alpha}}\newcommand{\bibeta}{\boldsymbol{\beta}}\newcommand{\bigamma}{\boldsymbol{\gamma}}\newcommand{\bidelta}{\boldsymbol{\delta}}\newcommand{\bivarepsilon}{\boldsymbol{\varepsilon}}\newcommand{\bizeta}{\boldsymbol{\zeta}}\newcommand{\bieta}{\boldsymbol{\eta}}\newcommand{\bitheta}{\boldsymbol{\theta}}\newcommand{\biiota}{\boldsymbol{\iota}}\newcommand{\bikappa}{\boldsymbol{\kappa}}\newcommand{\bilambda}{\boldsymbol{\lambda}}\newcommand{\bimu}{\boldsymbol{\mu}}\newcommand{\binu}{\boldsymbol{\nu}}\newcommand{\bixi}{\boldsymbol{\xi}}\newcommand{\biomicron}{\boldsymbol{\micron}}\newcommand{\bipi}{\boldsymbol{\pi}}\newcommand{\birho}{\boldsymbol{\rho}}\newcommand{\bisigma}{\boldsymbol{\sigma}}\newcommand{\bitau}{\boldsymbol{\tau}}\newcommand{\biupsilon}{\boldsymbol{\upsilon}}\newcommand{\biphi}{\boldsymbol{\phi}}\newcommand{\bichi}{\boldsymbol{\chi}}\newcommand{\bipsi}{\boldsymbol{\psi}}\newcommand{\biomega}{\boldsymbol{\omega}}\(\sigma \) is a parameter controlling the slope of the psychometric function. Ninety-five percent confidence intervals for individual size thresholds were obtained via nonparametric bootstrapping.

## Results

### Experiment 1: Motion improves masked visual acuity

**Figure 4:**
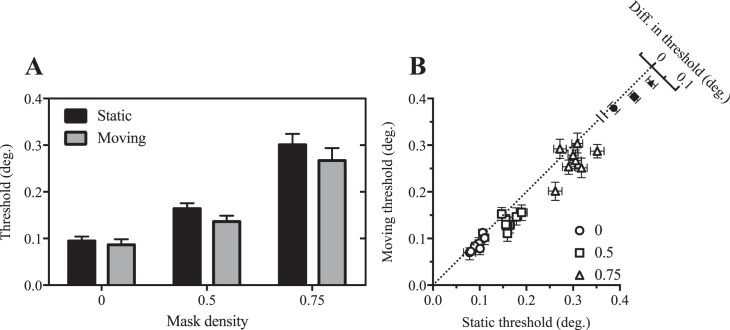
Motion improves masked visual acuity. (A) Mean size threshold for static (black) and moving (gray) targets as a function of mask density. Error bars show 95% confidence intervals. (B) Open symbols show data of individual observers separated by mask density; closed symbols show mean differences in size threshold between motion conditions (±95% confidence intervals), plotted on an oblique axis.

To extend the previous work of Frisén (2010) to the peripheral visual field, we first compared acuity for static and moving targets in the presence of static masks of varying density. Mean size thresholds are shown in Figure 4A. As expected, thresholds increase systematically as a function of mask density. Comparison of thresholds obtained with static and moving targets suggests that motion improved acuity, particularly when the target was masked. This motion-related benefit is also clearly visible in the bivariate scatter plot of individual subjects' thresholds shown in Figure 4B, where the majority of data points fall below the dashed diagonal line indicating equivalent performance in static and moving conditions.

To analyze these data, we first conducted a two-way repeated-measures analysis of variance (ANOVA). This revealed significant main effects of both mask density, *F*(2, 14) = 499.9, *p* < 0.0001, and target motion, *F*(1, 7) = 37.0, *p* = 0.0005, whereas the interaction between these factors approached significance, *F*(2, 14) = 3.2, *p* = 0.07. Decomposition of the interaction into simple effects indicated that target motion significantly improved performance for mask densities of 0.5, *t*(14) = 3.67, *p* = 0.003, and 0.75, *t*(14) = 4.49, *p* = 0.0005, but not 0, *t*(14) = 1.08, *p* > 0.05.

### Experiment 2: Residual motion-based improvement with dynamic mask updating

The results of Experiment 1 suggest that target motion is beneficial for acuity when performance is limited by undersampling of the stimulus but not by the natural sampling properties of the retinal periphery. However, because a static mask was used, the introduction of motion is confounded with an increase in the number of spatial samples available to the observer. To test whether motion provides any benefit beyond increasing the information content of the stimulus sequence, we next compared acuity for static and moving targets in the presence of randomly updated masks. This ensured that the number of independent target samples was matched in the two conditions and that any differences in performance could be directly attributed to motion of the target.

**Figure 5:**
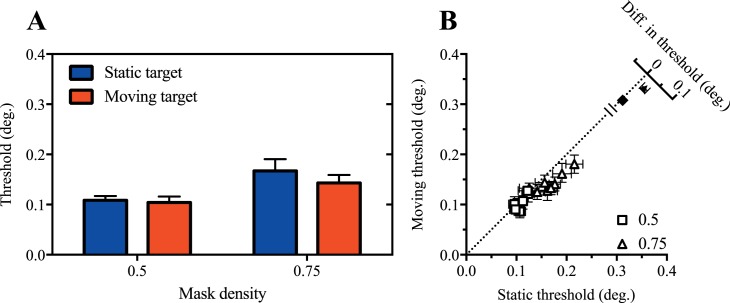
Residual motion-based improvement with dynamic mask updating. Mean (A) and individual (B) size thresholds are shown for static and moving peripheral targets behind masks with randomly updating element locations. Error bars show 95% confidence intervals.

As shown in Figure 5, under these conditions, motion produced a modest but consistent improvement in acuity. This effect was confirmed by the finding of a significant main effect of motion in a two-way ANOVA, *F*(1, 7) = 20.6, *p* = 0.003. We again found a significant main effect of mask density, *F*(1, 7) = 81.4, *p* < 0.0001, and in this case, the interaction between target motion and mask density was also significant, *F*(1, 7) = 9.6, *p* = 0.02. Analysis of the simple effects showed the effect of motion was significant in the 0.75 mask density condition, *t*(14) = 5.38, *p* = 0.001, but not 0.5, *t*(14) = 1.00, *p* > 0.05.

### Experiment 3: Target motion is more beneficial for visual acuity than mask motion

To investigate the specificity of motion-related acuity benefits, we next compared performance under conditions in which either the target moved behind a static mask or the mask moved in front of a static target. The same isoeccentric motion path and speed were used in both conditions.

**Figure 6:**
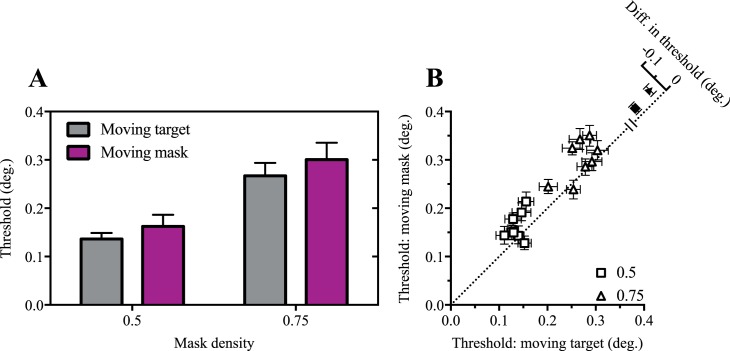
Target motion is more beneficial for visual acuity than mask motion. Mean (A) and individual (B) size thresholds for conditions in which either the target moved relative to a static mask or the target was static and presented behind a moving mask. Error bars show 95% confidence intervals.

Figure 6 indicates thresholds were lower for target motion than mask motion conditions, leading to a significant main effect of motion type in a two-way ANOVA, *F*(1, 7) = 8.9, *p* = 0.02. The ANOVA also indicated a significant main effect of mask density, *F*(1, 7) = 197.8, *p* < 0.0001, and no significant interaction, *F*(1, 7) = 0.6, *p* > 0.05. This indicates that the acuity benefits that arise from motion are specific to the target and not the mask.

### Experiment 4: Unpredictability in the motion path impairs visual acuity

In a final experiment, we investigated whether a smooth motion trajectory is required to support motion-related improvements. Random motion was generated by presenting the target at the same set of locations as in previous motion conditions but randomizing the presentation order of the frame sequence. Space-time plots of the smooth and random paths are depicted in Figure 3B and 3C, respectively.

**Figure 7:**
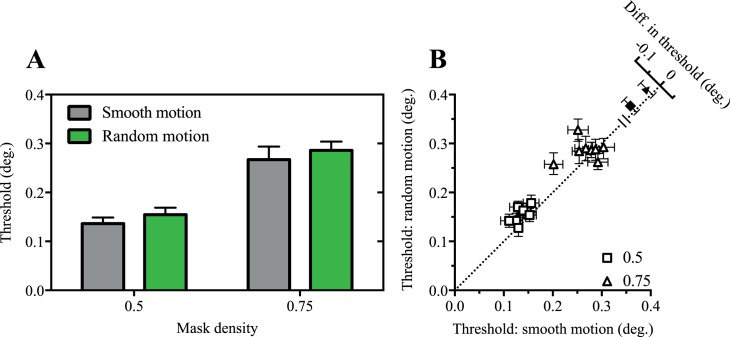
Randomizing the motion path impairs visual acuity. Mean (A) and individual (B) size thresholds for conditions in where the target either moved smoothly or randomly behind a static mask.

As shown in Figure 7, size thresholds were consistently lower in smooth motion than random motion path conditions. This was confirmed in a two-way ANOVA, where significant main effects of motion type, *F*(1, 7) = 11.6, *p* = 0.01, and mask density, *F*(1, 7) = 397.2, *p* < 0.0001, were found. The motion type × mask density interaction was not significant, *F*(1, 7) = 0.01, *p* > 0.05.

## General discussion

In this study, we sought evidence for the operation of motion-based SR mechanisms in the human periphery. In Experiment 1, we observed a statistically significant improvement in size thresholds for moving, compared with static targets viewed behind opaque masks. This is consistent with previous foveal studies of dynamic occlusion (Mateeff, Popov, & Hohnsbein, 1993; Palmer, Kellman, & Shipley, 2006; Shipley & Cunningham, 2001; Stevenson, Cormack, & Schor, 1989) and is a direct extension of Frisén's (2010) findings into the peripheral field. Although these benefits are consistent with the operation of a SR mechanism that integrates target information across space and time, it is important to note that when a target moves behind a static mask, more independent samples of the target are available in the stimulus sequence. Therefore, a stronger test of the SR hypothesis is to compare performance in static and moving conditions when stimulus information content has been matched. In Experiment 2, this was achieved by updating a dynamic mask, leading to a sizeable attenuation of the motion-related improvement. Accordingly, at least some of the effect of motion in Experiment 1, and presumably in the previous study by Frisén (2010), may be explained by the additional information available to the observer when forming a decision. Importantly, however, we found a significant residual motion-related benefit when stimulus information content was controlled. This provides more robust evidence for a dedicated motion-based SR mechanism for subsampled targets.

It is frequently observed that target motion is generally detrimental to spatial sensitivity; acuity drops quite dramatically as target speed is increased (Brown, 1972a, 1972b; Burr & Ross, 1982; Burr, Ross, & Morrone, 1986; Hammett, Georgeson, & Gorea, 1998; Westheimer & McKee, 1975), despite the operation of a dedicated deblurring mechanism (Burr, 1980; Hammett, 1997). However, Brown (1972b) showed that peripheral target resolution was slightly better for targets moving at 5°/s along the horizontal meridian than when static. This improvement in acuity at low speeds was not replicated in the present study where no opaque mask was applied to the target. One possibility for this discrepancy may lie in the methodological differences between the two studies: Rather than move stimuli along an isoeccentric path, Brown's manipulation allowed moving targets to encroach closer to the fovea than static targets. This encroachment may have been sufficient to yield an artefactual benefit in performance at low speeds.

There are a number of reasons why motion-based improvements in acuity may not be readily observable under normal peripheral viewing conditions. First, it may be the case that SR is dependent on the form and/or magnitude of the underlying image undersampling. Our masked conditions were designed to simulate loss of sampling units in the receptor array, by simultaneously obscuring multiple small, clustered regions of the target. Clustered photoreceptor degradation such as that resulting from retinal disease (e.g., cone-rod dystrophy; Hamel, 2007; Rabb, Tso, & Fishman, 1986) can have the effect of rendering a target only partially visible in this way. However, eccentricity-dependent changes in sampling are more akin to a progressive scaling of receptive fields. This scaling occurs because the spatial convergence of photoreceptors to retinal outputs layers changes dramatically across the retina. Indeed, measurements made using adaptive optics imaging and psychophysical testing suggest that, beyond the foveal center, spatial resolution is set by the properties of retinal ganglion cells in the output layer (Rossi & Roorda, 2010). SR mechanisms that operate by synthesizing image samples over time may be ill-suited to combating losses in acuity caused by this form of undersampling. Alternatively, the failure to find motion-related improvements in acuity could stem from difficulties in establishing a suitable baseline measure. Although performance in moving conditions was compared with conditions in which the target had a fixed location on the screen, this is not to say that there was no retinal motion. Even when subjects are asked to maintain steady fixation, there is natural drift of the image across the retina due to fixational eye movements (Martinez-Conde, 2006; Martinez-Conde, Macknik, & Hubel, 2004). Evidence suggests that this self-generated motion improves foveal acuity relative to situations in which images are stabilized on the retina (Ratnam, Domdei, Harmening, & Roorda, 2017). If it were the case that fixation instability is sufficient to engage SR mechanisms in the peripheral field, little or no additional benefit would be obtained by moving the target. Although we are not aware of any study that has directly compared peripheral visual acuity under stabilized and unstabilized conditions, computational accounts suggests that fixational eye movements might aid positional judgments across large regions of the visual field (Hennig & Wörgötter, 2004).

In Experiment 3, size thresholds were significantly lower when the target moved behind a static mask compared with the opposite situation in which the target is static and the mask moves. In the present study, the implementation of the opaque mask was intended to simulate the random loss of receptors in the underlying sampling array by partially obscuring parts of the image. Therefore, the two conditions represent situations in which there is object motion in visual space (target motion) or ocular motion (mask motion). Given that the motion of the target relative to the underlying sampling array is identical in each case, the asymmetry in size thresholds for these conditions appears paradoxical. However, because subjects were required to maintain fixation on a central marker throughout the trial, the moving mask condition, which would be akin to ocular motion, did not actually involve any movement of the eyes beyond the small jitter generated by fixational eye movements. The requirement to maintain fixation, therefore, created a situation in which there was a spatial decoupling between the retinal and simulated sampling array that was not present in any of the other conditions. The condition of a moving mask does not have a natural analogue that would exist when a visual scene is explored and as such may be unsuitable for engaging SR mechanisms. It is generally accepted that visual systems, whatever the species, are highly adapted to support the ecological needs of their owner. Visual functions are developed and refined by evolutionary processes to support repertoires of adaptive behaviors. Within this framework, it would be difficult to conceive of a mechanism that would exist for a situation an animal would never encounter in its natural environment, unless of course it was a by-product of another function. The presence of SR processing offers an acuity advantage to species that are able to exploit this motion-based information; we speculate that it may be limited in operation to previously encountered conditions.

For any form of SR processing to be possible, images obtained at successive points in time need to be co-registered with one another prior to synthesis. This requires that the system has access to the direction and speed of image motion (Park et al., 2003). In principle, this could be achieved in the brain via two mechanisms. First, when retinal motion is caused by movement of the eye, the system may have access to an efference copy of the motor command used to generate the eye movement (Bridgeman & Graziano, 1989). Although efference copy signals are thought to play important roles in visual processing (such as suppressing sensory processing of reafferent information), it is unlikely that they play a critical role in SR processing. Instead, recent findings suggest that similar improvements in foveal acuity are obtained regardless of whether or not retinal motion is congruent with fixational eye movements (Ratnam et al., 2017). The alternative approach is to estimate image motion directly and use estimates of the spatial shift between successive samples to achieve registration. Under this strategy, the success of SR will be dependent on the accuracy and precision of motion estimates. Motion coding is relatively trivial when objects move along smooth predictable trajectories but becomes more challenging when objects change position randomly over short time scales. This provides a potential explanation for the results of Experiment 4, in which acuity was found to be consistently better for targets moving along a smooth trajectory than those that moved unpredictably. In support of this, Mateeff and colleagues (1993) found that the visibility of simple geometric figures viewed through small pinhole apertures is improved when the figure moves smoothly compared to when it is presented at a series of random locations.

## Conclusions

Our results are consistent with the existence of an SR mechanism in the human periphery that combines information over space and time to improve visual acuity under conditions of simulated neural loss. We have shown that SR is most effective when the source of motion is the target and when the trajectory of motion is smooth and predictable. These findings may have practical implications for situations in which retinal disease leads to undersampling of the image. It has been proposed that SR processing uses fixational instability to compensate for acuity and sensitivity losses in eyes where retinal disease has caused dramatic changes to foveal cone structure (Ratnam, Carroll, Porco, Duncan, & Roorda, 2013; Ratnam et al., 2017). Combined with previous work, our findings raise the possibility that patients with neural loss affecting the central or near-peripheral visual field should benefit from the addition of smooth image motion.
